# HEPOM: Using Graph Neural Networks for the Accelerated Predictions of Hydrolysis Free Energies in Different pH Conditions

**DOI:** 10.1021/acs.jcim.4c02443

**Published:** 2025-04-04

**Authors:** Rishabh D. Guha, Santiago Vargas, Evan Walter Clark Spotte-Smith, Alexander Rizzolo Epstein, Maxwell Venetos, Ryan Kingsbury, Mingjian Wen, Samuel M. Blau, Kristin A. Persson

**Affiliations:** † Materials Science Division, Lawrence Berkeley National Laboratory, 1 Cyclotron Road, Berkeley, California 94720, United States; ‡ Chemical Sciences Division, Lawrence Berkeley National Laboratory, 1 Cyclotron Road, Berkeley, California 94720, United States; § Department of Materials Science and Engineering, Carnegie Mellon University, 5000 Forbes Avenue, Pittsburgh, Pennsylvania 15213, United States; ⊥ Department of Materials Science and Engineering, University of California, 210 Hearst Memorial Mining Building, Berkeley, California 94720, United States; # Department of Civil and Environmental Engineering, Princeton University, 86 Olden Street, Princeton, New Jersey 08544, United States; ¶ Institute of Fundamental and Frontier Sciences, University of Electronic Science and Technology of China, Chengdu 610054, China; ∇ Energy Storage and Distributed Resources, Lawrence Berkeley National Laboratory, Berkeley, California 94720, United States

## Abstract

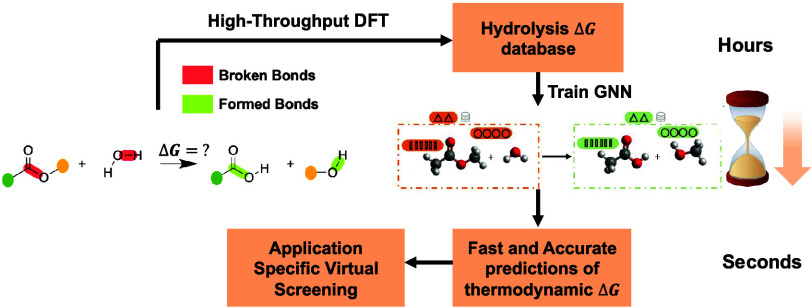

Hydrolysis is a fundamental family of chemical reactions where water facilitates the cleavage of bonds. The process is ubiquitous in biological and chemical systems, owing to water’s remarkable versatility as a solvent. However, accurately predicting the feasibility of hydrolysis through computational techniques is a difficult task, as subtle changes in reactant structure like heteroatom substitutions or neighboring functional groups can influence the reaction outcome. Furthermore, hydrolysis is sensitive to the pH of the aqueous medium, and the same reaction can have different reaction properties at different pH conditions. In this work, we have combined reaction templates and high-throughput ab initio calculations to construct a diverse data set of hydrolysis free energies. The developed framework automatically identifies reaction centers, generates hydrolysis products, and utilizes a trained graph neural network (GNN) model to predict Δ*G* values for all potential hydrolysis reactions in a given molecule. The long-term goal of the work is to develop a data-driven, computational tool for high-throughput screening of pH-specific hydrolytic stability and the rapid prediction of reaction products, which can then be applied in a wide array of applications including chemical recycling of polymers and ion-conducting membranes for clean energy generation and storage.

## 1 Introduction

Water is one of the most essential molecules in chemistry, and yet, its unique properties make it notoriously difficult to characterize.^1,2^ The significant electronegativity differences between its oxygen and hydrogen atoms gives water a highly polar character that leads to its recognition as the “universal solvent”.^3,4^ Hydrolysis, or any reaction where water acts as both a reactant and the solvent medium,^5,6^ are a prevalent class of reactions across chemistry. Hydrolytic reactions are fundamental in biological^7,8^ and synthetic chemistry^9,10^ and play a critical role in various essential scientific processes and significant technological applications. These range from processes, such as human digestion,^8,11^ where enzymes facilitate the hydrolytic breakdown of complex macronutrients into simpler compounds, to the degradation of hazardous pollutants^12^ and alternative plastic chemistries.^13^

At the molecular level, hydrolysis begins when a water molecule attacks specific sites on the reactant, initiating a sequence of bond cleavages and formations that lead to new product(s). The mechanism and the associated rate of this reaction is closely tied to the pH of the aqueous medium.^14,15^ The availability of protons (H^+^) or hydroxide (OH^–^) ions catalyzes the formation of charged species, which have markedly different reactivities compared to their neutral counterparts.^13,16^ These ionized reactants can exhibit enhanced solubility^17,18^ by forming stronger hydrogen bonds with the solvent. Additionally, water can act as catalyst, facilitating ion transfer through the solvent and creating alternate reaction pathways with lower energy barriers.^19,20^ As a result, acid/base-catalyzed hydrolysis of the same reactant can have significantly different reaction rates compared to its neutral form, adding complexity to the study of these reactions.

Given activation barriers (Δ*G*^⧧^), the experimental rate of a hydrolysis reaction can be directly correlated via the Eyring equation.^16,21,22^ This involves determining computationally intensive and difficult to find transition states for each individual reaction along the reaction coordinate of the potential energy surface (PES).^16,23,24^ In contrast, within a specific reaction family, the Bell–Evans–Polanyi principle (BEP)^25^ can offer an approximate linear correlation between the thermodynamic Gibbs free energy change (Δ*G*_r_) and the kinetic parameter Δ*G*^⧧^.^26−28^ In cases where it holds, BEP allows us to leverage the thermochemistry of products and reactants (Δ*G*_r_) to approximate trends in the kinetic rates of the reaction. This opens the avenue for the development of a computational screening tool that can calculate the respective Δ*G*_r_’s of all potential hydrolysis pathways and screen molecules for a specific hydrolysis-related application. Despite this, quantifying thermochemical quantities such as Δ*G*_r_ with high accuracy still requires DFT calculations with large basis sets and refined hybrid functionals at both reaction end points.^29,30^ Depending on the size of molecules, these calculations can take anywhere from several hours to days, particularly when employing implicit solvent models^31^ to approximate the contributions from the reaction environment.

Since computational cost is a severe bottleneck for any form of high-throughput screening, deep learning approaches have emerged as promising alternatives in the past decade, especially for tasks that involve the establishment of structure-to-property relationships.^32,33^ Recently, graph convolutions, which iteratively update node and edge features based on connectivity and local environment, have proven to be extremely effective in learning molecular^34,35^ and reaction representations.^36,37^ Despite these methodological advances, the largest roadblock to the development of an accurate model is typically the procurement of diverse, representative data. For instance, the model developed by Grambow et al.^33^ was facilitated by a data set of 12,000 gas-phase reactions^38^ sampled from a subset of molecules in the GDB-17 data set.^39^ The bond dissociation energy (BDE) prediction framework developed by Wen et al.^40^ was trained on a data set of over 60,000 homolytic and heterolytic bond dissociation reactions.^41^ In the realm of hydrolysis, no such comprehensive data set currently exists.

In this work, we have attempted to address these shortcomings by first developing a predictive framework based on reaction templates for different functional groups that can automatically generate hydrolysis products for multiple pathways in any molecule. This framework was then applied on a subset of the QM9^42^ and the Alchemy^43^ databases to generate a database of over 65,000 hydrolysis reactions in an implicit aqueous solvation environment. For a given reactant molecule in the QM9 subset of the data, we have also generated corresponding hydroxylated and protonated states of the reactant molecule to approximate the effects of extreme pH on the Δ*G*_r_ of hydrolysis. In addition, the neutral fold of the data set was developed with reactants from the QM9 database and later augmented with the inclusion of larger reactant molecules from the Alchemy^43^ data set. Combined, we provide a new data set hydrolysis reactions that encompasses thermodynamic properties at different charged(protonated/hydroxylated) states along with a large, exploratory set of neutral-pH reactions for analysis and model development.

We then proceeded to use this comprehensive data set to train a GNN model, which serves as a **H**ydrolysis **E**nergy **P**redictor for **O**rganic **M**olecules (**HEPOM**). The model leverages the difference features of the atom (node), bond (edge), and global features between the products and the reactants to directly predict the DFT-calculated Δ*G*_r_. The global reaction atom mapping allows the model to simultaneously track multiple bond dissociations and formations. For the neutral data set, the model achieved a low mean absolute error (MAE) of 1.73 kcal/mol on a diverse holdout set of hydrolysis reactions and it was also successful in outperforming a diverse set of benchmark models on the smaller and more complex protonated and hydroxylated data sets.

## 2 Methods

### 2.1 Reaction Generation

**Figure 1:**
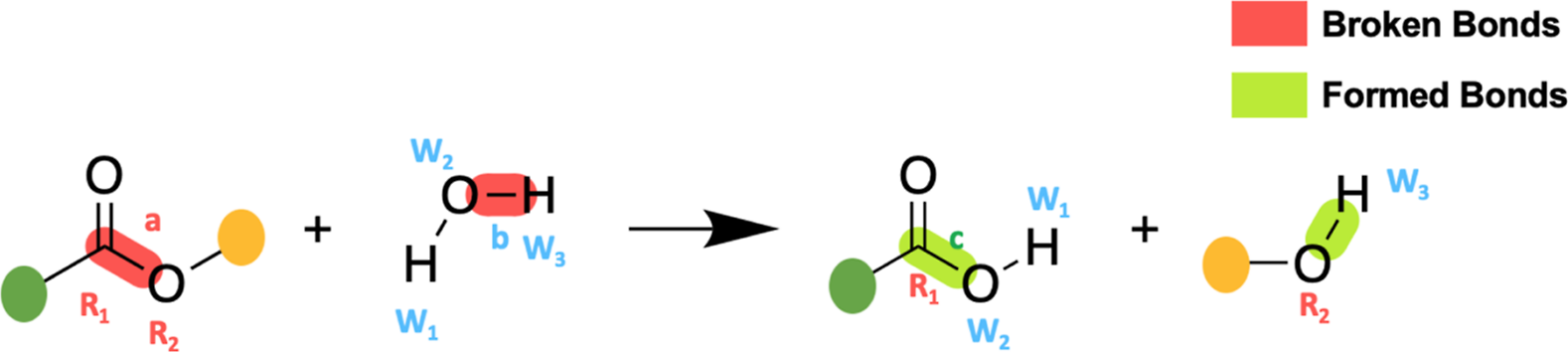
Set of bond cleavage and formations necessary to generate hydrolysis products for a representative ester molecule.

We segmented the construction of our data set into four main parts: three derived from the QM9 data set (representing neutral, protonated, and hydroxylated reactions) and another, neutral reaction set from the Alchemy data set. Hydrolyzable molecules in QM9 were screened using RDKit^44^ substructure matching for 20 standard, hydrolyzable functional groups (Figure S5b). These templates were adapted from the work by Tebes-Stevens et al.^45^ and integrated into an automated framework to predict reaction products. For instance, in Figure 1, if an ester functional group was detected in a molecule, the reaction template would yield a carboxylic acid and an alcohol as the respective hydrolysis products. Bond ‘a’ in the reactant and bond ‘b’ in the water molecule were deleted with the RemoveBond functionality in RDKit. Then, AddBond was used to create bonds ‘c’ and ‘d’ between atoms R1-W2 and R2-W3 respectively, to yield a carboxylic acid and an alcohol as the respective products. Similar reaction templates were implemented for all functional groups. If multiple competing functional groups were identified in a single molecule, then the hydrolysis products were generated independently for each individual functional group (Schematic S1 in the Supporting Information) and added as separate reaction entries to the database.

Nitriles were treated differently: the reaction template yields amides and these can be further hydrolyzed into an amine and a carboxylic acid (Schematic S2 in the Supporting Information). As a result, the intermediate products of nitrile reactions served as reactants in additional hydrolysis reactions, thereby augmenting the data set. We generated a total of **16,264** hydrolysis reactions from the QM9 data set. An initial model was trained on **15,264** of these neutral reactions, while **1000** reactions were kept in an unseen holdout test set. Additional details regarding the product generation workflow has been included in Section S3 of the Supporting Information. The performance of this model is discussed in Section 3.2 (*vide infra*).

The broader goal of this work is to develop a framework capable of enumerating potential hydrolysis pathways for a wide range of molecules and predicting the thermodynamic free energies of these pathways with high accuracy. With this in mind, we screened molecules from the Alchemy data set^43^ to generate data set entries representing larger, more complex reactions. The Alchemy data set includes molecules with up to 14 heavy atoms, though we only considered molecules with more than 10 heavy atoms. Neutral reactions generated from the Alchemy data set were added to the original data set, resulting in a combined data set of **41,006** reactions. Of these, **2800** reactions were filtered out and added to the previous QM9 test set, creating an unseen holdout test set of **3800** reactions (approximately 10% of the training set size). In the following sections of the manuscript, we refer to the original data set as the QM9 data set and the expanded data set as the QM9 + Alchemy data set.

While the data generated above are useful and novel, they are examples of hydrolysis in a neutral reaction medium. However, hydrolysis is often catalyzed in an acidic or basic reaction medium. For example, the hydrolysis rate of amides in a neutral medium is negligible, even after heating, but amidic hydrolysis proceeds at a moderate rate in an acidic or basic medium,^46,47^ forming carboxylic acid and an amine. Consequently, we explored whether this framework could extend to broader reaction conditions, potentially serving as a screening tool for identifying molecules amenable to pH-specific hydrolysis. It is important to clarify that under these reaction conditions, hydrolysis is initiated by the protonation or hydroxylation of the reacting functional group,^20,48^ and the overall reaction rate is heavily influenced by the *pK*_a_ values of the functional groups.^49,50^ Generating a diverse data set in high throughput for acid- or base-catalyzed hydrolysis while accounting for *pK*_a_ was intractable with our current data generation scheme. Therefore, in this work, we focused our efforts on developing a unified model that can predict the differences in hydrolysis reaction-free energies for the same functional group under different pH conditions. The data sets generated for neutral or basic pH assume that the reaction medium is at an extreme pH, i.e., if a functional group can protonate or hydroxylate, it will. An alternative approach could involve applying a separate ML model which predicts the *pK*_a_ and identifies the most probable site for protonation or hydroxylation at a specific pH,^51−53^ but this is beyond the scope of the present study.

We separated extreme pH hydrolysis reactions into two reactions schemes. For an acidic medium, the reacting functional group was assumed to be protonated at the most electron-rich atom site (e.g., the carbonyl oxygen in an ester or amide, or the nitrogen atom in a nitrile). Conversely, for a basic pH, the functional group’s relevant electrophilic site—such as the carbonyl carbon in an ester or amide or the ring carbon in the epoxide—was hydroxylated. The acidic pH reaction was then executed between the protonated reactant and two water molecules to maintain reaction stoichiometry. A representative example elucidating the differences in the hydrolysis reaction in acidic and neutral pH for a hydrolyzing carbamate molecule is demonstrated in Figure S4a,b of the Supporting Information. The extra water molecule on the reactant side absorbs the proton to generate hydronium as one of the reaction products. This approach circumvents the erroneous DFT-calculated energies of an isolated proton in an implicit solvent medium.^54^ In the case of basic pH, the hydroxylated reactant decomposes into the reaction products and a hydroxide ion. For these two data sets, we focused on the QM9 molecules to limit the scope of computations, yielding a protonated data set of **11,323** reactions and a hydroxylated data set of **16,732** reactions. Holdout test sets consistent with the neutral QM9 data set were also extracted before model training. Since the protonated reactants have a +1 charge and the hydroxylated reactants a –1 charge, we will refer to these data sets as QM9^+^ and QM9^–^, respectively, in the subsequent sections.

### 2.2 Density-Functional Theory

QChem (version 5 or 6)^55^ was used to perform all the DFT calculations necessary to generate the data set. A specialized frequency-flattening optimization (FFOpt) workflow, originally developed by Spotte-Smith et al.^41^ and currently implemented in atomate^56^ was used to optimize the reactant and product structures to a true minima and also obtain thermochemical quantities from the vibrational frequencies. The workflow iteratively performs successive geometry optimizations and frequency calculations until there are either none or a single negligible negative frequency (<15 cm^–1^). This approach ensures that the optimized structure is a true local minimum of the PES and not a saddle point. Moreover, the workflow parses the necessary enthalpy and entropy terms from the QChem frequency output document for the free energy calculations. For all the DFT calculations, we used the range-separated metaGGA hybrid functional, ωB97M-V,^57^ which employs the VV10 dispersion correction,^58^ to improve the noncovalent interactions. The def2-SVPD basis set^59^ was employed for the FFOpt workflow and the solvation effects were implicitly accounted for with the water SMD solvent model.^17^ The electronic energies of the optimized structures were refined with single-point calculations using a larger def2-QZVPPD basis set.^59^

### 2.3 Model Architecture

**Figure 2:**
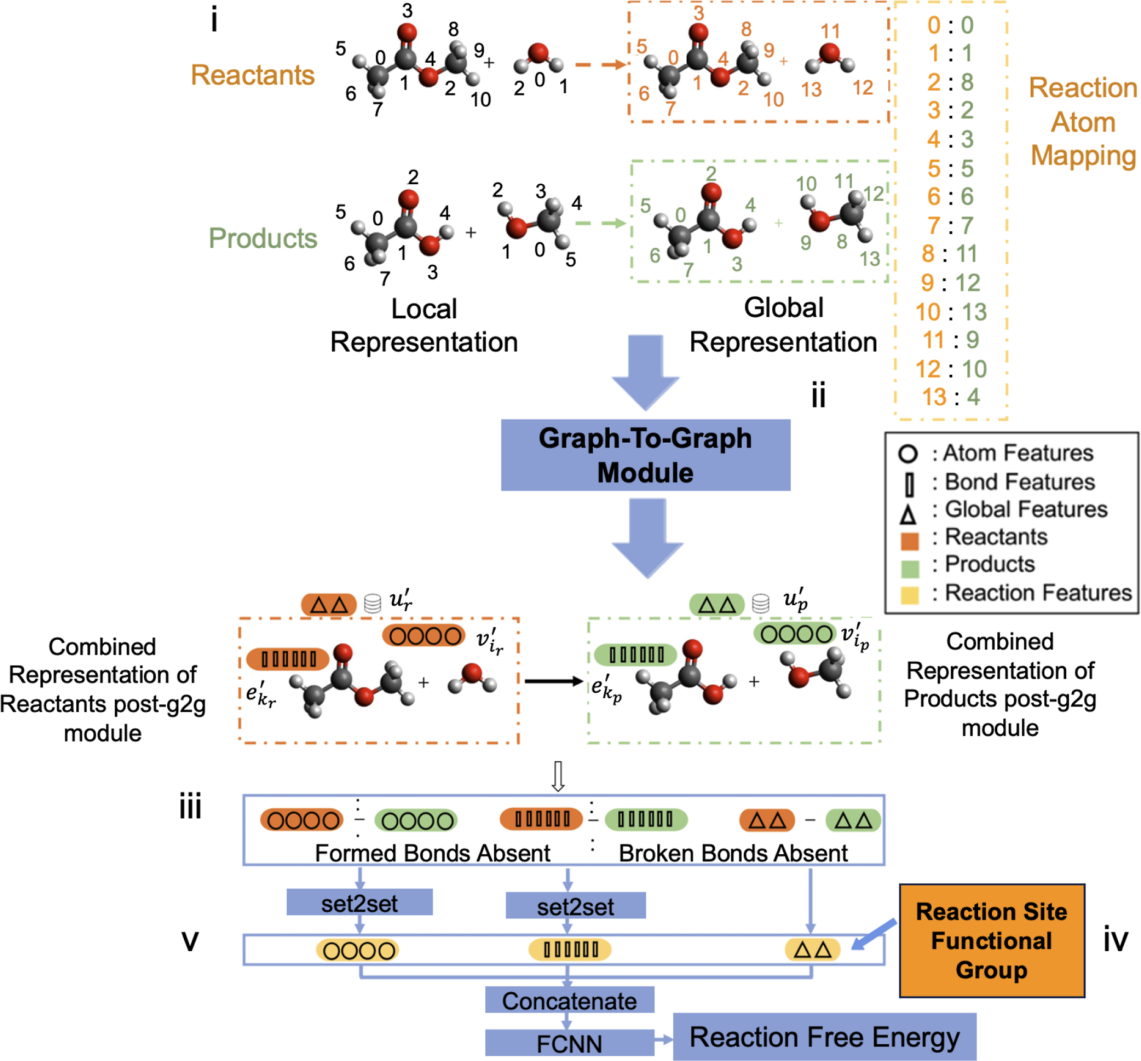
GNN Architecture: The user inputs atom-mapped sets of reactants and products (i) which undergo message-passing and update steps (ii). Using the user-specified mappings, these updated features are mapped to a global reaction graph (iii) where functional groups are the reaction site is added as a global feature (iv). Embeddings of bond and atom features plus global features directly serve as the fixed-size vector used in a conventional dense neural network for property prediction.

The GNN model(Figure 2) is based on the previous BonDNet architecture.^40^ Here we briefly discuss that model before highlighting our key departures and how these differences are key to working with our data sets here.

The original algorithm uses gated graph convolutional (GatedGC) layers to propagate initial node features within the graphs of individual species on both sides of a reaction. While GatedGC layers were used widely for structure-to-property models in chemistry and materials science,^60,61^ BonDNet improved on these previous implementations by integrating update and message-passing equations between global nodes and atom/bond type nodes; this allows for the treatment of species of different charges and provides a framework to include molecular-level features. Similar to other graph neural networks, more distant graph relationships are treated by iteratively stacking several (typically 2–4 layers) GatedGC layers. With updated species graphs, reaction graphs are built to hold reaction feature differences—atom and bond nodes are mapped to each other on both sides of a reaction and their features are subtracted between corresponding atoms/bonds. Broken bonds are represented by zero vectors in this scheme. Here BonDNet implemented a custom set2set^62^ global pooling feature to map updated reaction graphs to fixed-sized vectors. These vectors are passed to fully connected layers for property prediction.

Our implementation extends pooling by integrating a diverse set of global pooling functions, including set2set,^62^ WeightedMeanPooling, Self-attention pooling,^63^ and Mean Pooling. This diverse set of global pooling functions was intended to provide a more comprehensive toolkit of architectures across different data set sizes. Previous benchmarks showed set2set layers did not always outperform simpler MeanPooling approaches.^64^

In this implementation, the reaction mapping is altered from the original BonDNet to a global reaction graph that is constructed between the union set of bonds in products and reactants. Originally, BonDNet used the product graph as a scaffold and subtracted reactant features from corresponding nodes in the product graph. This limited the model to only being applicable for *A* → *B* and *A* → *B* + *C* type reactions with a single bond dissociation. The previous framework could not interpret a hydrolysis reaction that involves at least two elementary bond dissociation and formation reactions. Our algorithm builds a global reaction graph by taking the union set of atoms and bonds in products and reactants and uses this to build a graph structure with bonds from each side of reaction. The approach allows us to precompute global graphs, including mappings and descriptors, for offline preprocessing prior to training. This change allows for an arbitrary number of bond changes, simultaneous breaking and forming, to be treated by the model (Figure 2). In addition, we are able to generalize our model to any number of species on either side of the reaction—a feature critical for hydrolysis where no reaction can fit BonDNet’s original implementation. This update is important for others looking to use our new model for reaction-property prediction as now the model is completely flexible to any number of bond changes and number of species.

For the task of hydrolysis, where we have a consistent reaction framework, we incorporated a one-hot encoding of functional group identity^45^ into the global feature nodes. This encoding provides a simple, yet effective, descriptor that captures the reaction site of hydrolysis reactions alongside the more distant features generated by stacked message-passing layers. This is a particularly attractive feature, as sequential stacking of message-passing layers rapidly increases compute time and can lead to problems such as oversmoothing.^65,66^ Our heterograph architecture, by including global nodes connected to every atom and bond, allows for distal information passing while avoiding such issues. We also implemented a host of computational features such as multiGPU compatibility, a pytorch-lightning implementation, and added support for preprocessing data sets. These latter features are vital for our data sets while also providing the community with a flexible, general GNN architecture for reaction-property prediction at scale.

## 3 Results and Discussion

### 3.1 Data Set Overview

As detailed in Section 2.1, our hydrolysis database, in its current form, comprises a total of **68,761** reactions, making it the largest molecular database for hydrolysis reactions. Among these, the QM9 + Alchemy data set contains **41,006** reactions with reactant molecules in their neutral state, while the remaining reactions are approximately evenly split between the QM9^+^ (protonated) and QM9^–^ data sets, representing acidic and basic pH conditions, respectively.

The QM9 + Alchemy data set contains reactants with up to 12 heavy atoms. The distribution of reactants based on the number of heavy elements is illustrated in Figure S5c of the Supporting Information. For the charged subsets (QM9^+^ and QM9^–^), the reactants are restricted to a maximum of 9 heavy atoms.

The number of hydrolyzed products varies depending on the reacting functional group, with reactions yielding one, two, and, in some cases (e.g., urea and carbamates), three products. Figure S5a of the Supporting Information visualizes the distribution of reactions based on the number of products generated, and Figure S5b illustrates the distribution across different hydrolyzed functional groups.

**Figure 3:**
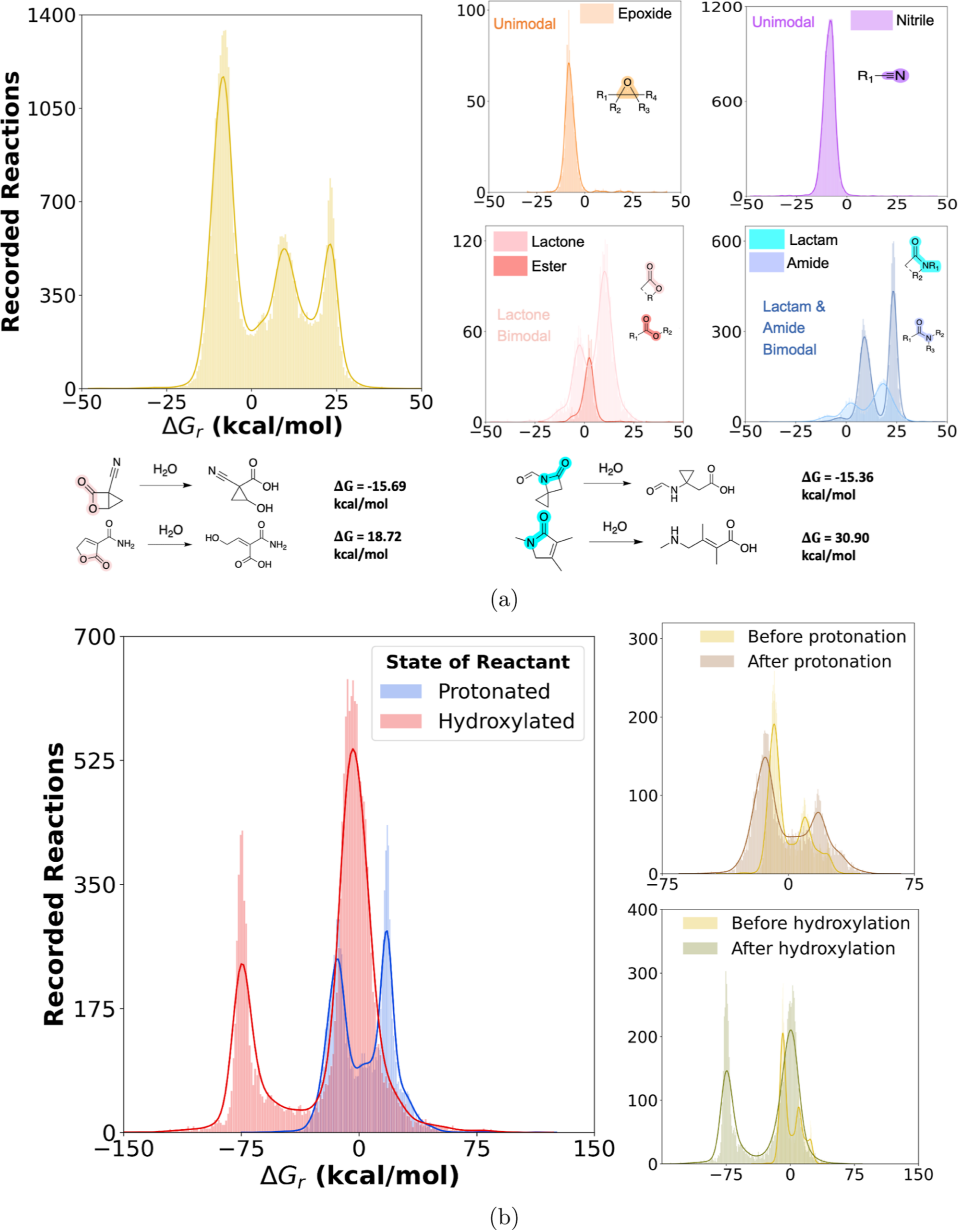
(a) Distribution of hydrolysis Δ*G*_r_ for the QM9 + Alchemy data set. The associated subfigures show the Δ*G*_r_ distributions for specific functional groups (b) Distribution of hydrolysis Δ*G*_r_ for the QM9^+^ and QM9^–^ data sets. The associated subfigures show the shift in the Δ*G*_r_ distributions for a subset of common reactants in the three data sets. The *x* and *y* axes on all the subfigures represent the same metrics as the main figures.

The hydrolysis Δ*G*_r_ distribution for the QM9 + Alchemy data set is presented in Figure 3a, where three peaks are observed: two distinct peaks in the endergonic region (Δ*G*_r_ > 0) and one larger peak in the exergonic regime (Δ*G*_r_ < 0). Interestingly, the Δ*G*_r_ distribution in Figure 3a is almost perfectly balanced with **20,547** reactions (50.11%) of the neutral reactions falling within the endergonic regime.

Further analysis across different functional groups reveals some interesting insights. Distributions of epoxides, nitriles, and esters exhibit unimodal energy distributions, while cyclic esters and cyclic amides (e.g., lactones and lactams) are bimodal. Sampling random lactone and lactam reactions (Figure 3a) from the endergonic and exergonic regimes indicates that cyclic structures with strained rings have more favorable thermodynamic hydrolysis pathways, whereas stable five-membered rings are more resistant to hydrolysis.^67,68^ The hydrolysis of amides shows a distinctly bimodal nature, with both peaks centered in the endergonic regime, consistent with the established trend of the thermodynamic infeasibility of amide hydrolysis in a neutral reaction medium.^69^

The energy distribution for the protonated (QM9^+^) and hydroxylated (QM9^–^) data sets is shown in Figure 3b. It is evident that the Δ*G*_r_ distribution for hydroxylated reactants shifts strongly toward the exergonic regime with greater than 70% of the reactions with a thermodynamically exergonic hydrolytic pathway. The shift in the protonated data set is more subtle; however, when comparing the corresponding slices of the same reactions in the neutral and protonated states (Figure 3b), it is clear that the distribution broadens after protonation.

The following section will discuss our model’s performance on these different data sets and how it compares to existing benchmarks.

### 3.2 Model Performance—Neutral Data Set

In the initial round of model training, we utilized a data set of hydrolysis reactions generated exclusively from reactants extracted from the QM9 database. Despite a modest training set of **15,264** reactions, the model performed well on the holdout test set of **1000** reactions across ten different functional groups. The mean absolute error (MAE) was **2.44 kcal/mol**. Further details on the model’s performance with this smaller data set are provided in Section S6 of the Supporting Information.

**Figure 4:**
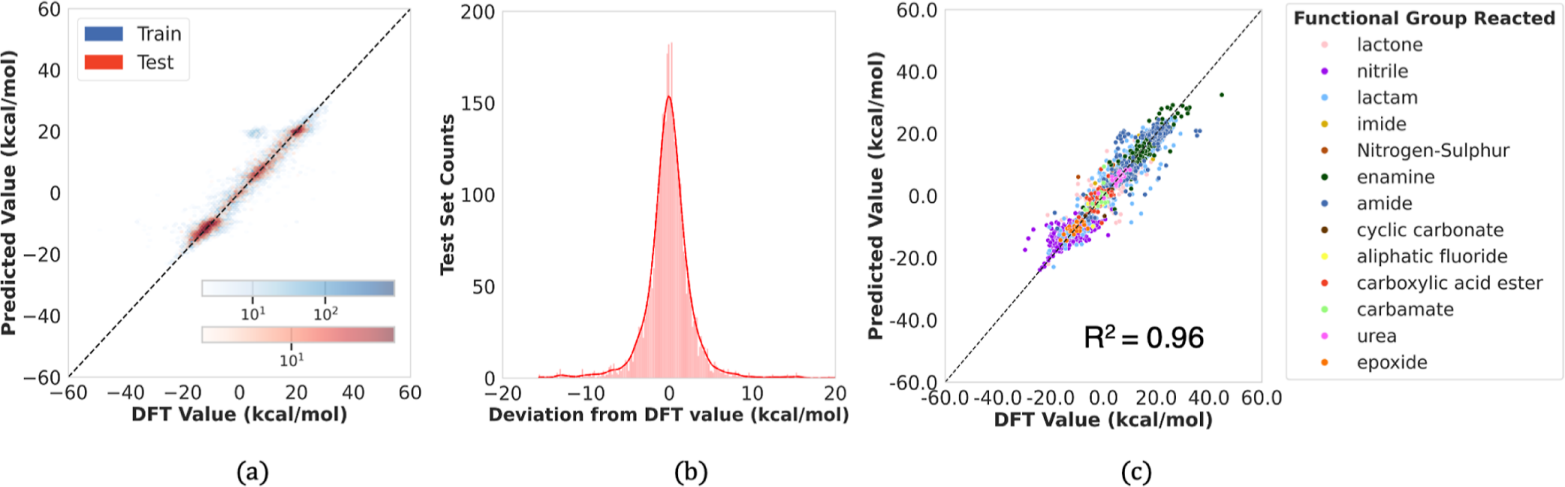
Performance of HEPOM on the QM9 + Alchemy data set. (a) Δ*G*_r_ predicted by HEPOM versus DFT reference labels for the train and test sets; (b) histogram of the prediction errors; (c) parity plot for the holdout test set segregated on the basis of hydrolyzed functional group.

Compared to other studies using molecular GNNs for property prediction,^35,40,70^ this training data set is relatively small. However, to the best of our knowledge, there are no publicly available data sets specifically for hydrolysis reactions. To evaluate the impact of additional training data, we curated **24,742** more reactions from the Alchemy database, focusing on molecules with 10, 11, or 12 heavy atoms. This expansion also increased the variety of hydrolyzing functional groups from 10 to 13. The expanded data set was randomly split into training and testing sets at roughly a 9:1 ratio, resulting in a holdout test set of **3800** reactions. As shown in Figure 4a, the model generalized effectively on this test set, with the MAE improving to **1.73 kcal/mol**. The parity plot comparing model predictions with DFT labels for the test set, shown in Figure 4c, demonstrates a high coefficient of determination (*R*^2^) of **0.96**. The distribution of deviations between model predictions and DFT labels, illustrated in Figure 4b, indicates that errors are closely centered around a mean of zero kcal/mol. A detailed breakdown of these errors is provided in Table 1, showing that only 53 out of 3800 reactions had prediction errors exceeding 10 kcal/mol, thus suggesting the model is suitable for screening purposes in this regime. Section S7 of the Supporting Information includes examples of five such outlier reactions, which often feature unusual structures with multiple strained rings, potentially contributing to the larger prediction errors. Another important aspect of evaluating the model’s applicability is its capability to classify the overall thermodynamic feasibility of hydrolysis reactions as either exergonic or endergonic, based on the DFT labels. The model correctly classifies 97.1% of the reactions in the test set, demonstrating its strong predictive power in distinguishing between these two thermodynamic outcomes. Among the 117 misclassified reactions, a significant proportion (72) had DFT-calculated Δ*G*_r_ values close to zero, highlighting the inherent difficulty of correctly classifying these reactions.

**Table 1:** Error Distribution for the QM9 + Alchemy Holdout Test Set

absolute error (kcal/mol)	counts
<2	2674
>2 and <5	893
>5 and <10	180
>10	53

Table 2 summarizes MAE statistics by functional group, revealing that no specific functional group performs poorly. Notably, functional groups like lactones and lactams, which exhibit a broader range of Δ*G*_r_ as shown in Figure 3a, tend to have higher MAEs. The higher MAE for imides may be attributed to their lower representation in the database (165 out of 41,006 reactions). However, interestingly, some functional groups with lower representation, such as aliphatic fluoride (191) and cyclic carbonate (116), show lower MAEs compared to the model average.

**Table 2:** MAE Statistics Based on Functional Group Hydrolyzed

functional group	MAE (kcal/mol)
lactone	2.198
nitrile	1.433
lactam	2.408
imide	2.498
nitrogen–sulfur cleavage	2.176
enamine	2.098
amide	1.724
cyclic carbonate	1.252
aliphatic fluoride	1.022
carboxylic acid ester	1.436
carbamate	1.591
urea	1.091
epoxide	1.571
overall average	1.731

**Figure 5:**
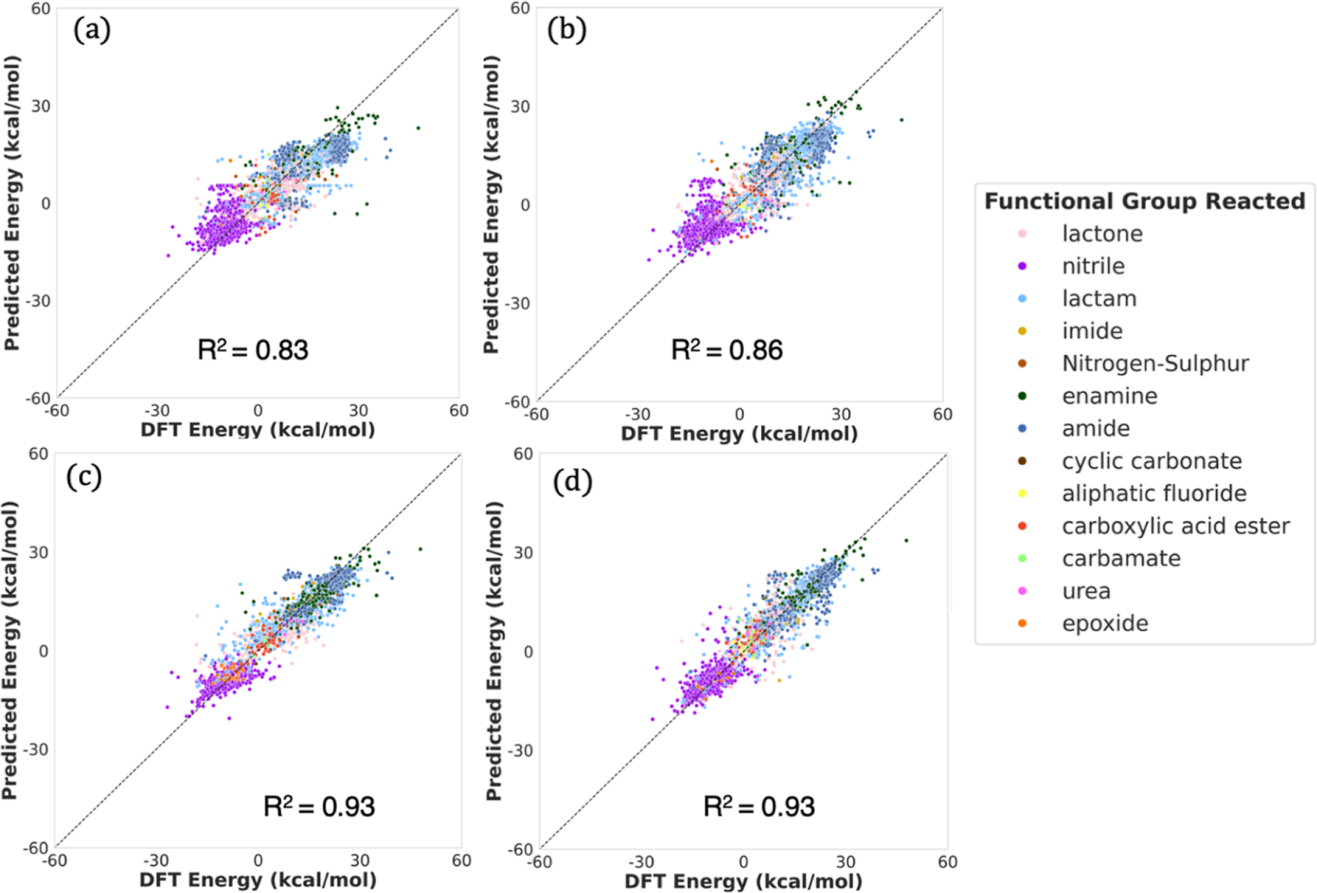
Parity plots for the performance of benchmark models on the holdout test of the QM9 + Alchemy data set. (a) Reactant only GNN—node features; (b) reactant only GNN—node + edge features; (c) XGBoost + Morgan fingerprints; (d) Chemprop.^70^

To assess our model’s performance relative to other reaction-property prediction algorithms, we benchmarked it against several models. As detailed in Section 2.3, our model is highly generalizable and capable of handling reactions with varying numbers of bond changes—a feature not commonly found in reaction-property algorithms. This limitation narrowed the range of models suitable for benchmarking. We tested a simple reactant-only graph neural network with both atom and bond features, incorporating standard chemoinformatics features such as bond degree, element identity, atomic weight, ring inclusion, and hybridization, as well as global features such as the number of atoms and bonds, molecular weight, and one-hot encoding for the hydrolyzing functional group and charge. Additionally, we evaluated an XGBoost model with Morgan Fingerprints and Chemprop,^70^ another modern algorithm. Both the XGBoost and Chemprop models were tuned using Bayesian optimization before final testing. The performance of these models is summarized in Table 3. Although Chemprop performs competitively (MAE: 2.25 kcal/mol vs 1.73 kcal/mol), our model outperforms all benchmarked models in terms of performance metrics on the holdout test set. The performance of individual benchmark models is shown in Figure 5.

**Table 3:** Performance Comparison against Benchmark Models for QM9 + Alchemy Dataset^a^

model	test MAE (kcal/mol)	test RMSE (kcal/mol)
mean	12.745	14.670
reactant GNN (atom)	4.008	5.429
reactant GNN (atom + bond)	3.445	4.875
XGB + morgan	2.448	3.705
chemprop	2.257	3.528
*HEPOM*	1.731	2.674

a We also include a benchmark to trivially guessing the mean of the training set.

### 3.3 Model Embeddings—Neutral Data Set

Visualizing the feature space provides insight into the underlying patterns the model learns during training. To analyze the learned representations for the trained model, we extracted high-dimensional difference feature vectors for each test set reaction before they are implemented into the fully connected layer for prediction. These vectors were then reduced to a two-dimensional (2D) space using the Uniform Manifold Approximation and Projection (UMAP) method.^71^ The evolution of these 2D embeddings at different epochs during training is shown in Figure S8 of the Supporting Information.

**Figure 6:**
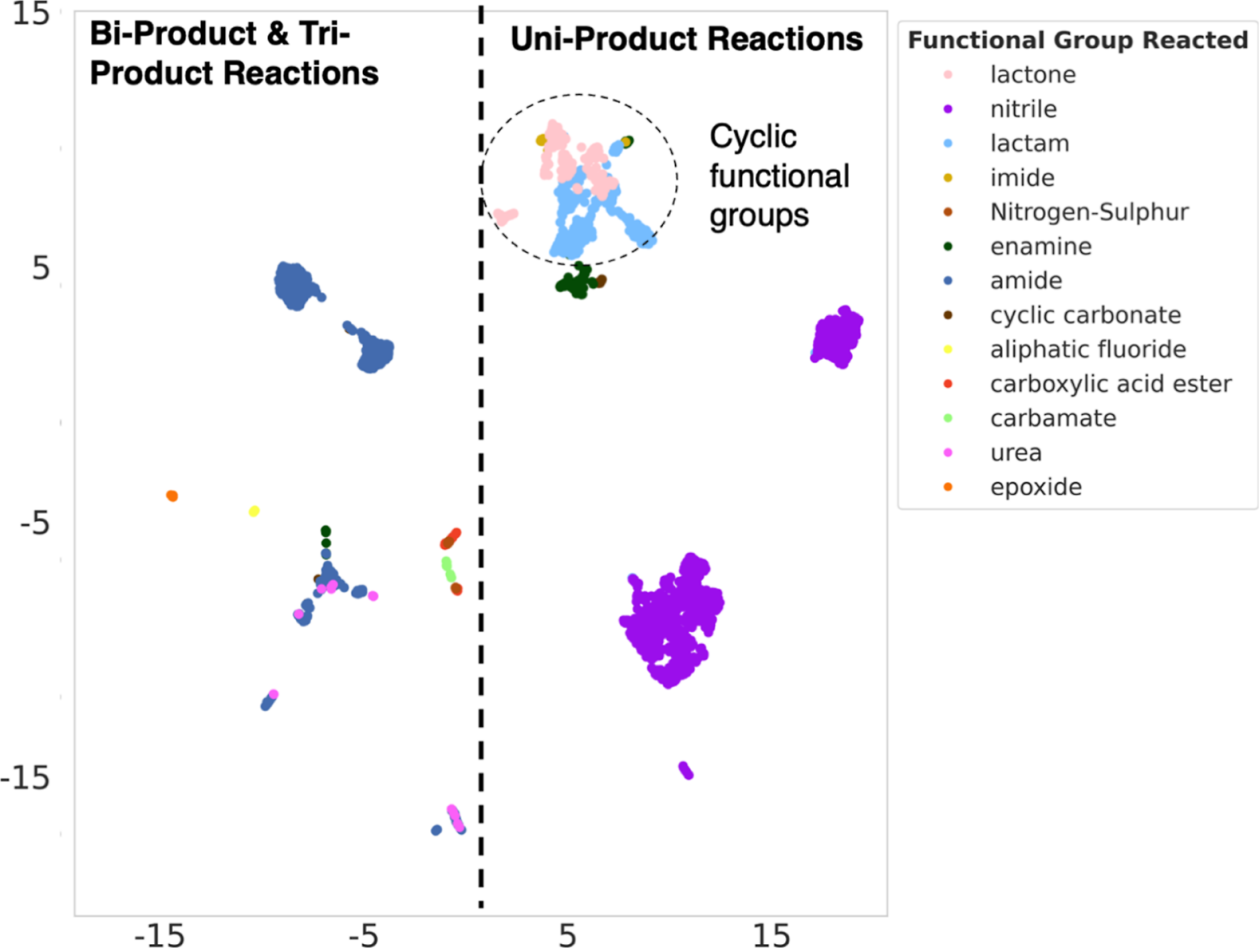
UMAP embeddings of the high-dimensional feature vectors representing the hydrolysis reactions into a two-dimensional space.

Initially, the embeddings are loosely clustered based on the functional groups of the hydrolyzing reactants, as expected. However, as training progresses, these clusters become tighter and more defined, reflecting not only functional groups but also other underlying chemical similarities not explicitly known to the model. Figure 6 illustrates the final 2D representations of the feature vectors for the test set, each tagged with its respective hydrolyzing functional group. In addition to clustering by functional groups, a clear distinction emerges between the embeddings of uniproduct reactions and those of biproduct and triproduct reactions. Uniproduct reactions predominantly cluster on one side of the feature vector space, while reactions yielding more than one product aggregate oppositely. For uniproduct reactions, the model forms a distinct cluster for cyclic functional groups (lactams, lactones, and imides). This suggests that the model identifies additional common features beyond functional group similarity, such as ring-opening during hydrolysis.

### 3.4 Model Performance: QM9^+^ and QM9^–^ Data Sets

**Figure 7:**
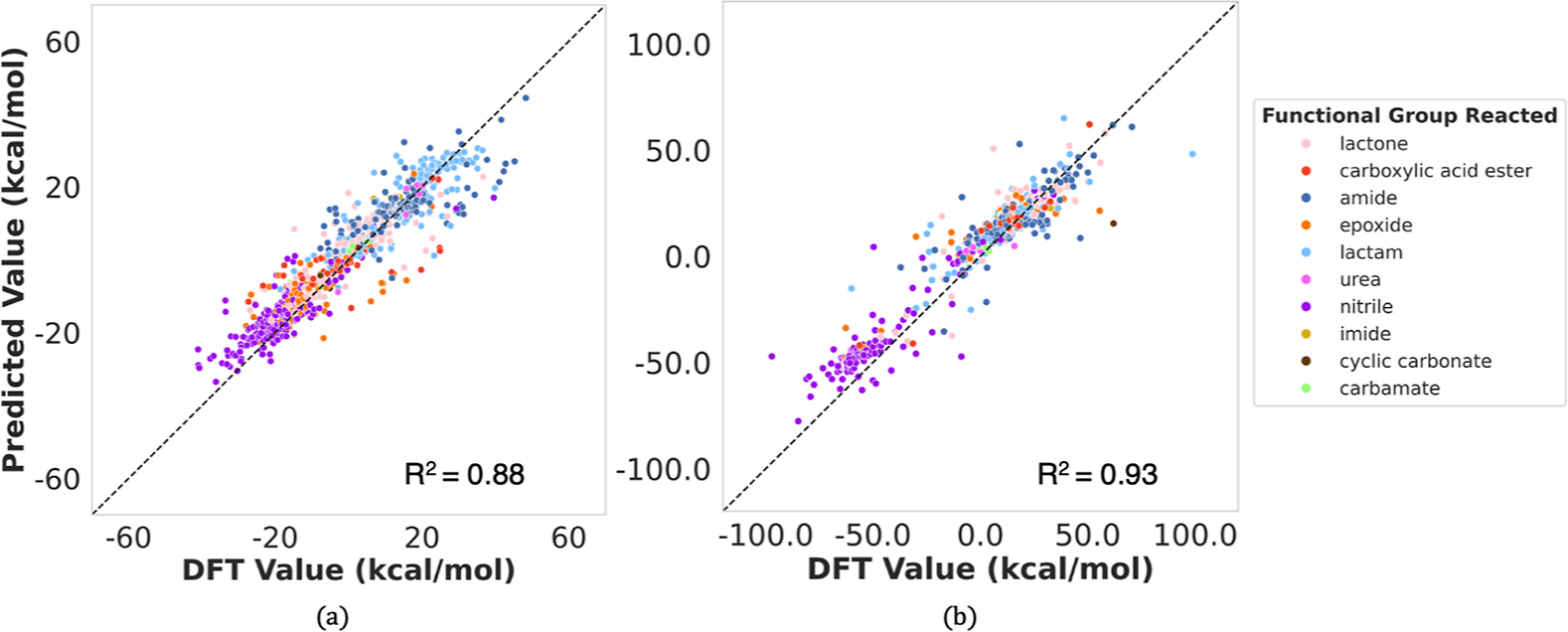
Test Set Performance on models trained with the (a) QM9^+^ protonated and (b) QM9^–^ hydroxylated data sets.

In Section 2.1, we discussed that in most practical scenarios, hydrolysis occurs in a reaction medium where acidic or basic pH expedites the reaction. To extend the model’s applicability, we generated the QM9^+^ and QM9^–^ data sets, which include protonated and hydroxylated reactants, respectively, to simulate extreme pH conditions. In the current work, the protonation/hydroxylation was limited to only the QM9 molecules. Therefore, these charged data sets are considerably smaller than the neutral QM9 + Alchemy data set. The trained models’ performance on the holdout test sets is shown in Figure 7 and summarized in Table 4.

**Table 4:** Performance Comparison against Benchmark Models for QM9^+^ and QM9^–^ Datasets, Best Model Is Bolded

model	**QM9**^**+**^**data set**		**QM9**^**–**^**data set**	
	test MAE **(kcal/mol)**	test RMSE **(kcal/mol)**	test MAE **(kcal/mol)**	test RMSE **(kcal/mol)**
mean	15.234	17.381	34.831	36.919
XGB + Morgan	5.394	8.195	8.687	14.375
chemprop	6.275	8.864	**5.373**	9.682
*HEPOM*	**4.282**	**6.213**	6.607	**9.326**

As expected, the model’s performance on these data sets deteriorates, evidenced by the lower coefficient of determination (*R*^2^) and higher mean absolute errors (MAEs) for both the QM9^+^ and QM9^–^ test sets. This performance decrease is particularly pronounced for the hydroxylated model, which shows a relatively high MAE of 6.607 kcal/mol. However, it is important to contextualize this result by noting that the range of Δ*G*_r_ values in this data set is radically different, roughly spanning between −150 and 100 kcal/mol, compared to the QM9 + Alchemy data sets (−40 to 40 kcal/mol). Notably, the MAE value also corresponds to a strong *R*^2^ of 0.93.

Given these differences, a fair comparison of model performance should be made with relevant benchmarks rather than the neutral data set. For these two data sets, we conducted hyperparameter optimization using XGBoost and Chemprop models. As shown in Table 4, our model outperforms the benchmarks in most metrics in this smaller and more complex data set. The exception is the mean absolute error (MAE) on the QM9^–^ data set, where Chemprop slightly outperforms HEPOM. Interestingly, despite the higher MAE for HEPOM on the QM9^–^ test set, it demonstrates greater robustness to outlier predictions, achieving a lower root mean squared error (RMSE) compared to Chemprop. Furthermore, HEPOM significantly outperforms Chemprop in correctly classifying the thermodynamic feasibility (endergonic vs exergonic) of reactions in the QM9^–^ data set, achieving a classification accuracy of 95.5% compared to Chemprop’s 84.8%. XGBoost has the lowest classification accuracy of 76.3% on the same test set. Additional details, including parity plots for the benchmarks and MAE statistics by functional group for these holdout test sets, are provided in Section S9 of the Supporting Information.

### 3.5 Combined Model Training

**Figure 8:**
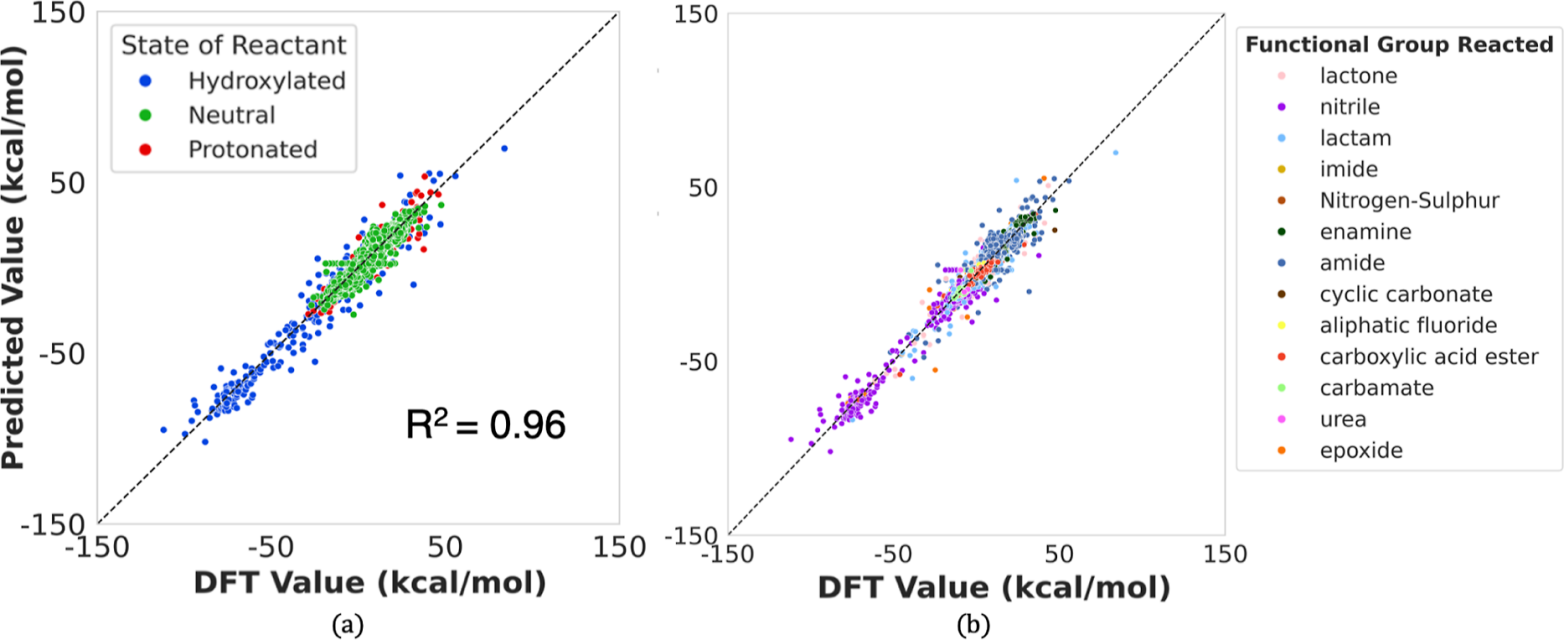
Test Set Performance on models trained with the combined data set depicted as (a) the charged state of the reactant and (b) the hydrolyzing functional group.

For the neutral data set, we observed a significant improvement in model performance after incorporating additional data from the Alchemy data set. Given this result, it can be expected that the performance of the protonated (QM9^+^) and hydroxylated (QM9^–^) models would also benefit from more data. However, obtaining this would require another round of computationally intensive data curation. Instead, we chose to augment the data sets by combining all three data sets. We found that adding the neutral data significantly improved the performance of both charged models, as shown in Figure 8.

Importantly, this improvement was not limited to our models but was also observed in the benchmark models, particularly Chemprop. This suggests that the higher MAEs of the QM9^+^ and QM9^–^ models may be simply due to insufficient data. However, as seen in Table 5, the most pronounced improvement was in our HEPOM model, which allowed it to surpass the other benchmarks, including in the previously higher MAE for the QM9^–^ test set (Table 4).

**Table 5:** Performance Comparison against Benchmark Models the Combined Dataset, Best Model Is Bolded

model	test MAE (kcal/mol)	test RMSE (kcal/mol)
XGB + Morgan	9.998	14.756
chemprop	4.920	8.562
*HEPOM*	3.054	4.281

The parity plots in Figure 8a and the MAE split based on the reactant state, compiled in Table 6, show that the improved model performance for the charged data sets comes at the cost of a slightly higher MAE for the neutral test set. The corresponding parity plots for the benchmark models are included in Section S10 of the Supporting Information.

**Table 6:** Our Model MAE Stratified on the Combined Test Set Based on the Reactant State

state of reactant	mean absolute error (kcal/mol)
hydroxylated	4.057
neutral	2.805
protonated	2.838

## 4 Conclusion

In this work, we combined reaction templates and high-throughput DFT calculations to generate a large and diverse data set of Δ*G*_r_ values of hydrolytic pathways for molecules selected from two popular molecular databases (QM9 and Alchemy). We then used this data set to train a custom message-passing GNN on the difference features of the products and reactants, resulting in a model capable of predicting the thermodynamic feasibility (Δ*G*_r_) of hydrolysis reactions. The model demonstrates remarkable accuracy on the neutral data set of hydrolysis reactions and outperforms benchmark models on smaller, more complex data sets involving charged reactants, simulating extreme pH conditions. In addition, by combining all three of our data sets, we find that our model is able to reasonably predict across all three classes at once.

We believe that this model is valuable for high-throughput screening of molecules and automated chemical synthesis in various domains, including drug development, environmental chemistry, and chemical deconstruction. The comprehensive data set developed in this work also serves as a critical resource for training other machine learning models. In terms of the model, although this study focuses on hydrolysis, the model can be easily fine-tuned for any arbitrary reaction data sets with available reactant and product molecule graphs.

Training and holdout test sets for all models are publicly accessible via Figshare, and detailed information about the reactant and product molecules for the QM9 database is available via the MPCules^72^ interface, with future plans to integrate the Alchemy data set reactants and products as well. The code for training the model can be accessed at the GitHub repository.

## Data Availability

The training and holdout test sets for all models are publicly available via Figshare: https://figshare.com/articles/dataset/Hydrolysis_Datasets_from_HEPOM_paper_/27851130.The code for training the model is available on the HEPOM GitHub repository: https://github.com/HEPOM/HEPOM. Model hyperparameters for each of the trained models are available in Section S11 of the Supporting Information

## Supporting Information Available

Additional details about data set generation, data set statistics, model performance against benchmarks, and model hyperparameters (PDF)
