# Supplementary material for: HEPOM: Using Graph Neural Networks for the Accelerated Predictions of Hydrolysis Free Energies in Different pH Conditions
Source: J Chem Inf Model. 2025 Apr 4;65(8):3963–75. doi: 10.1021/acs.jcim.4c02443 (PMC12042266; doi:10.1021/acs.jcim.4c02443)
Supplement: Supplementary file 1 — ci4c02443_si_001.pdf [file ci4c02443_si_001.pdf]

Supporting Information for: HEPOM: Accelerating the Prediction of Hydrolysis Free Energies Across Different pH Conditions Using Graph Neural Networks

Rishabh D. Guha,[†,‡,△] Santiago Vargas,[¶,△] Evan Walter Clark Spotte-Smith,[§] Alexander Rizzolo Epstein,[‖] Maxwell Venetos,[†,‖] Ryan Kingsbury,[⊥] Mingjian Wen,[#] Samuel M. Blau,[@] and Kristin A. Persson[*,‖,†]

†*Materials Science Division, Lawrence Berkeley National Laboratory, 1 Cyclotron Road, Berkeley, CA, 94720 USA*

‡*Present Address: Schrödinger Inc. 1540 Broadway, New York, NY 10024, USA*

¶*Chemical Sciences Division, Lawrence Berkeley National Laboratory, 1 Cyclotron Road, Berkeley, CA, 94720 USA*

§*Department of Materials Science and Engineering, Carnegie Mellon University, 5000 Forbes Ave., Pittsburgh, PA, 15213 USA*

‖*Department of Materials Science and Engineering, University of California, Berkeley, 210 Hearst Memorial Mining Building, Berkeley, CA, 94720 USA*

⊥*Department of Civil and Environmental Engineering, Princeton University, 86 Olden Street, Princeton, NJ 08544*

#*Institute of Fundamental and Frontier Sciences, University of Electronic Science and Technology of China, Chengdu, 610054, China*

@*Energy Storage and Distributed Resources, Lawrence Berkeley National Laboratory*

△*These authors contributed equally to this work*

E-mail: kristinpersson@berkeley.edu

Table of Contents

S1. Schematic S1: Competing Hydrolyzable Functional Groups **S2**

S2. Schematic S2: Dataset Augmentation **S3**

S3. Section S3: Granular Details on Product Generation from `RDKit` Reactants **S4**

S4. Schematic S4: Neutral pH vs. Acidic pH Hydrolysis **S5**

S5. Section S5: Insights on the Neutral Hydrolysis Database **S6**

S6. Section S6: Model Performance on the `QM9`-Only Dataset **S9**

S7. Section S7: Examples of Extreme Outliers in the Test Set **S10**

S8. Section S8: Evolution of the 2D Feature Embeddings with Training Epochs **S11**

S9. Section S9: Benchmark Comparison and Additional Performance Details for the $\texttt{QM9}^{+}$ and the $\texttt{QM9}^{-}$ Datasets **S12**

S10. Section S10: Benchmark Comparison and Additional Performance Details for the Combined Datasets **S15**

S11. Section S11: Model Hyperparameters **S15**

Schematic S1: Competing Hydrolyzable Functional Groups

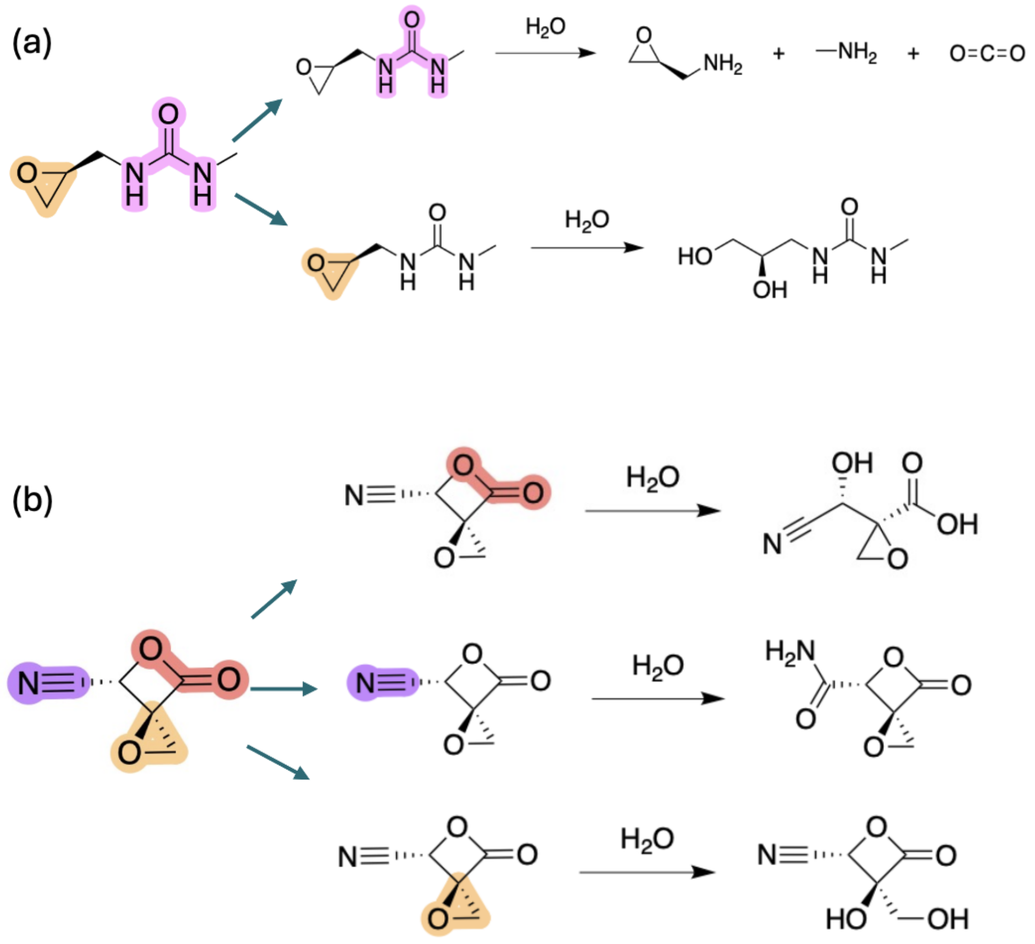

Figure S1: Schematic representation of the process for generating multiple reactions when competing functional groups are detected in a reactant: (a) Two functional groups detected and (b) Three functional groups detected.

Schematic S2: Dataset Augmentation

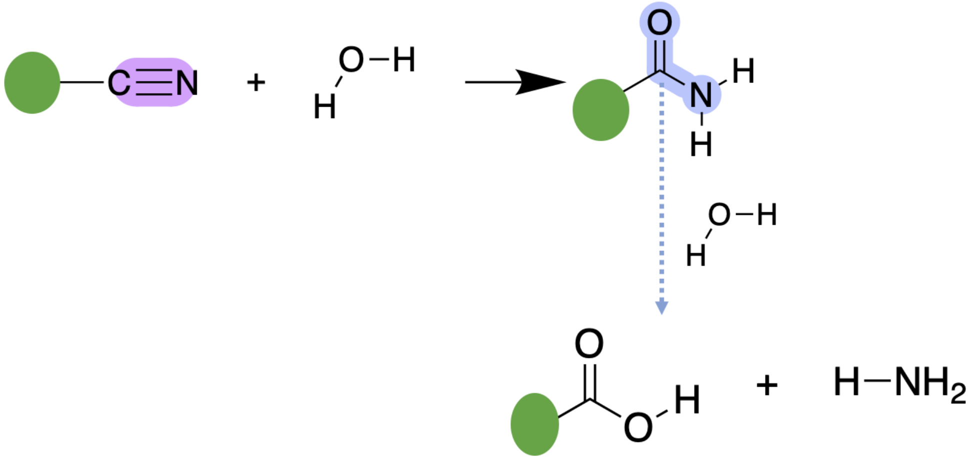

Figure S2: Amide reactions from the products of nitrile hydrolysis.

Section S3: Granular details on product generation from `RDKit` reactants

1. Reactant Molecules and SMILES Strings:

- In this work, the SMILES strings were used only as identifiers for reactant molecules in the database. These SMILES strings can be accessed from the `reactant_smiles` column in the database.
- To construct RDKit molecule representations of reactants, we directly processed the `.xyz` files available in the QM9 and Alchemy datasets using the `xyz2mol` package, which transformed the files into RDKit molecule objects.
- Canonical SMILES strings for the reactants were generated using RDKit's `Chem.MolToSmiles` function and served solely as identifiers. These were not used for generating 3D geometries.
- Hydrolyzable functional groups were detected by directly processing the reactant RDKit molecules, and all molecular editing was performed on these RDKit molecule representations.

2. Generating Product Fragments and Storing 3D Geometries:

- For each identified hydrolysis pathway, RDKit was used to create molecules corresponding to the product fragments, which were later converted to 3D geometries using a three-step process:
  (a) The product fragments were first converted into intermediate SMILES representations with RDKit's `Chem.MolToSmiles` functionality.
  (b) The SMILES strings were then used to create `OpenBabel` molecule objects in Python via the `pybel` package. OpenBabel's `addh()` function was used to add hydrogen atoms, and its `make3D()` function was used to generate a 3D geometry.

(c) Finally, the generated 3D `OpenBabel` molecules were converted into `pymatgen` molecule representations using the `BabelMolAdaptor` class from the `pymatgen.io.babel` module. These `pymatgen`[1] molecules, representing the 3D geometries of the product fragments, were stored in the database for downstream processing and model training.

To ensure no unphysical reactions or erroneous geometries were included in the dataset, we performed strict filtering after the DFT optimizations of each reaction entry. Specifically, any reactions where reactants or products underwent simultaneous bond formation and breaking during optimization were excluded from the database. The complete code of the product generation process is available in our GitHub repository at: `HEPOM/bondnet/Hepom_dataset_development/heuristic_hydrolysis_products.py`.

Schematic S4: Neutral pH vs. Acidic pH Hydrolysis

a. In neutral pH

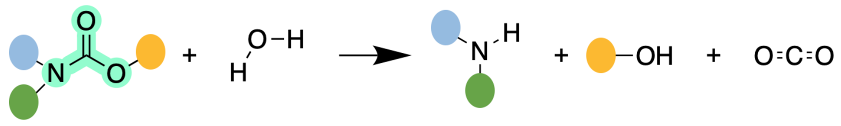

b. In highly acidic pH

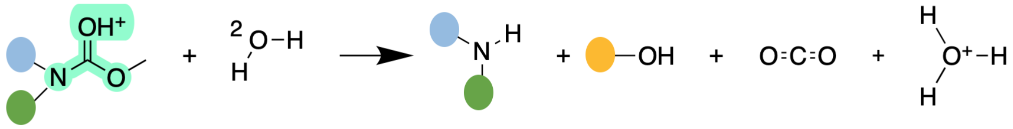

Figure S4: Hydrolysis reaction template for a representative carbamate molecule in (a) Neutral pH and (b) Highly acidic pH conditions.

Section S5: Insights on the neutral hydrolysis database

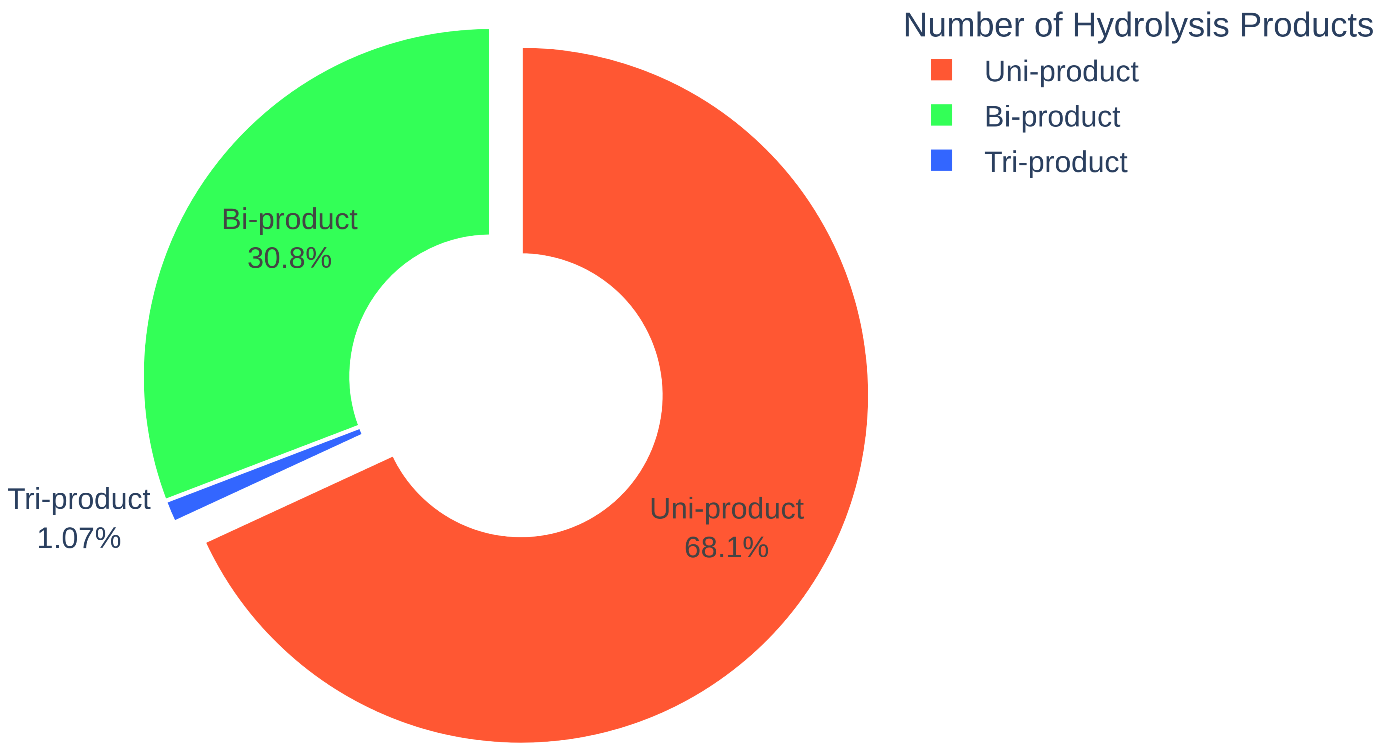

Figure S5(a): Distribution of hydrolysis reaction types based on the number of products generated.

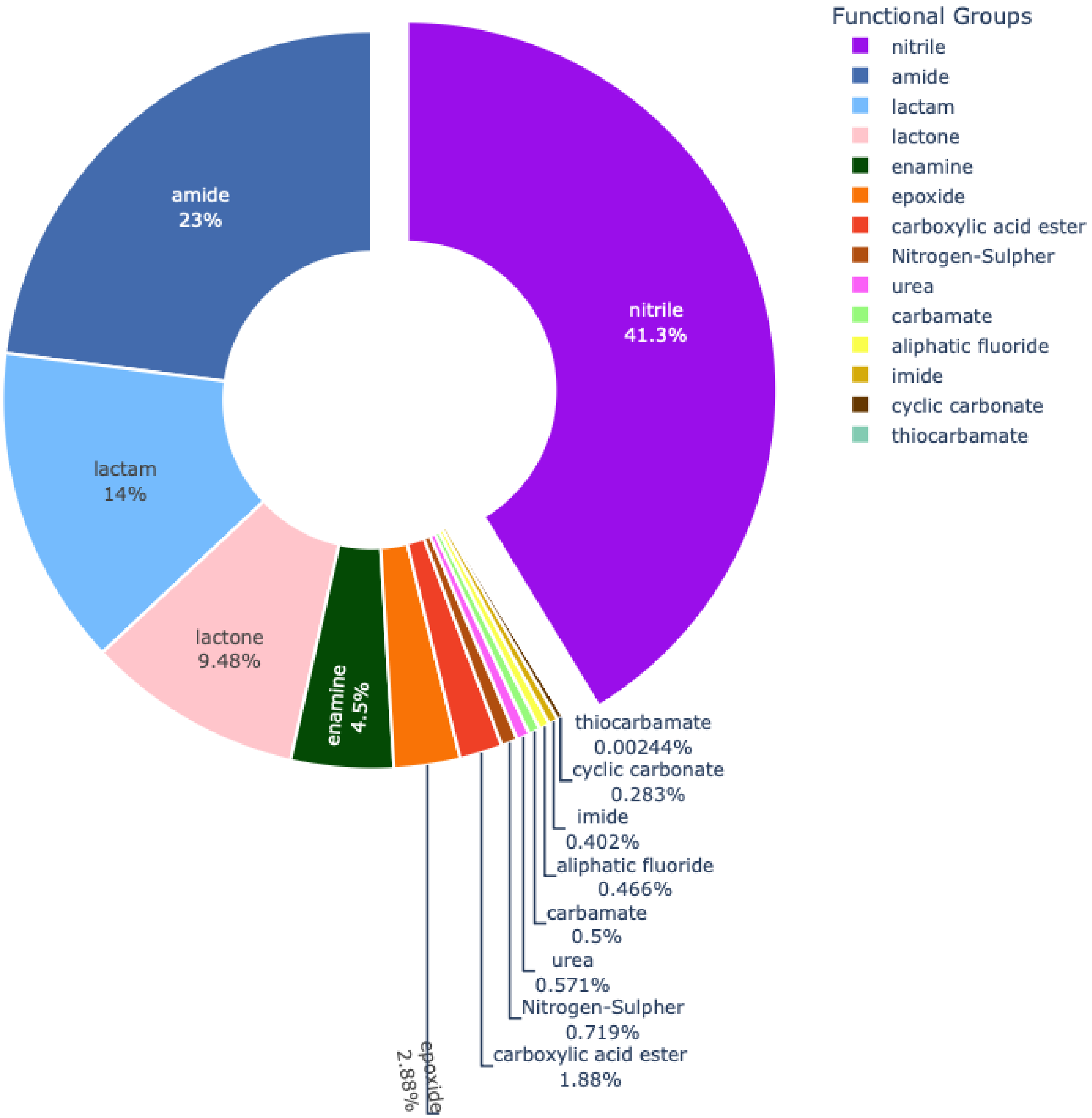

Figure S5(b): Distribution of hydrolysis reactions based on the hydrolyzing functional group.

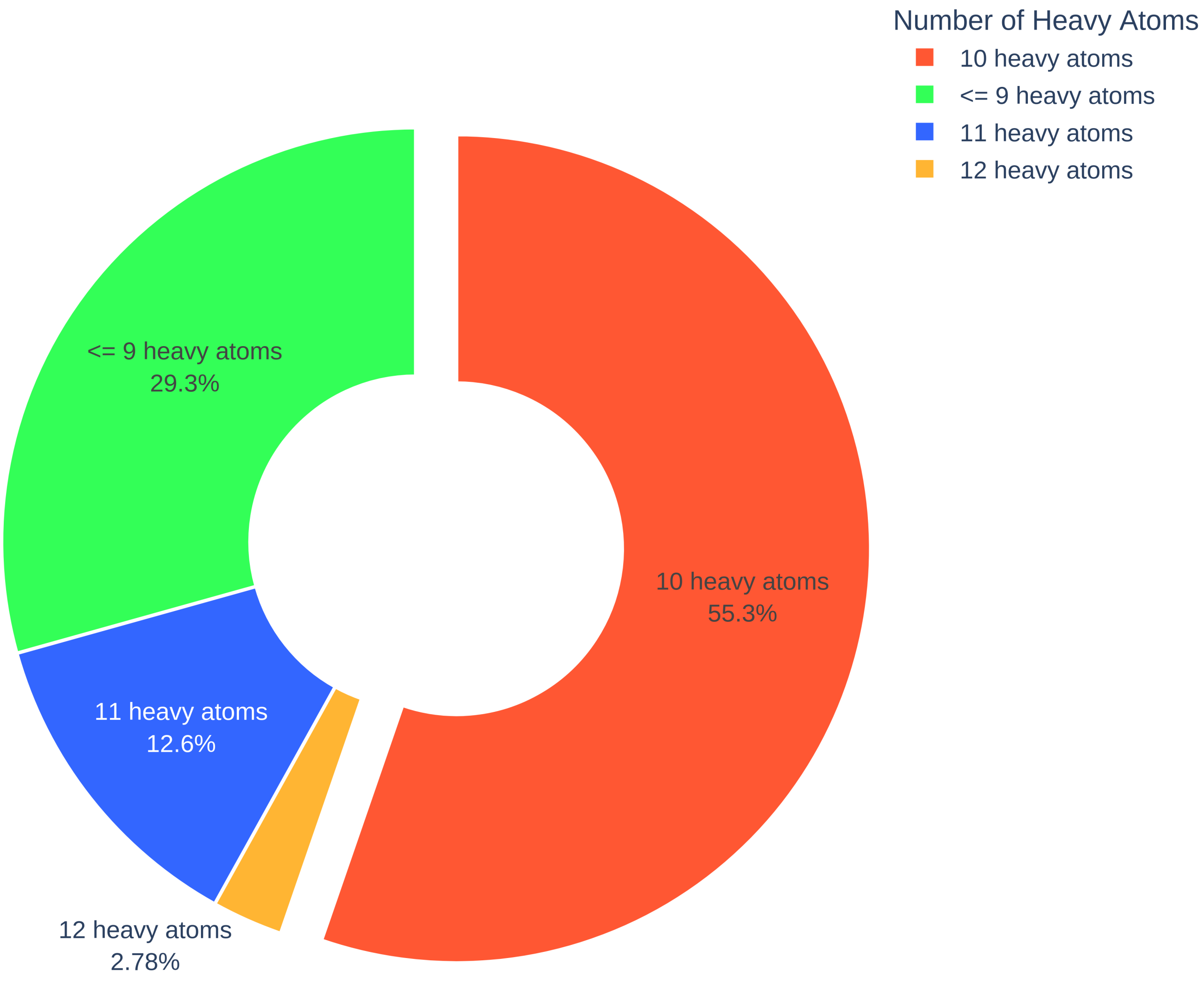

Figure S5(c): Distribution of hydrolysis reactions based on the number of heavy atoms in the reactant.

Section S6: Model Performance on the QM9-only dataset

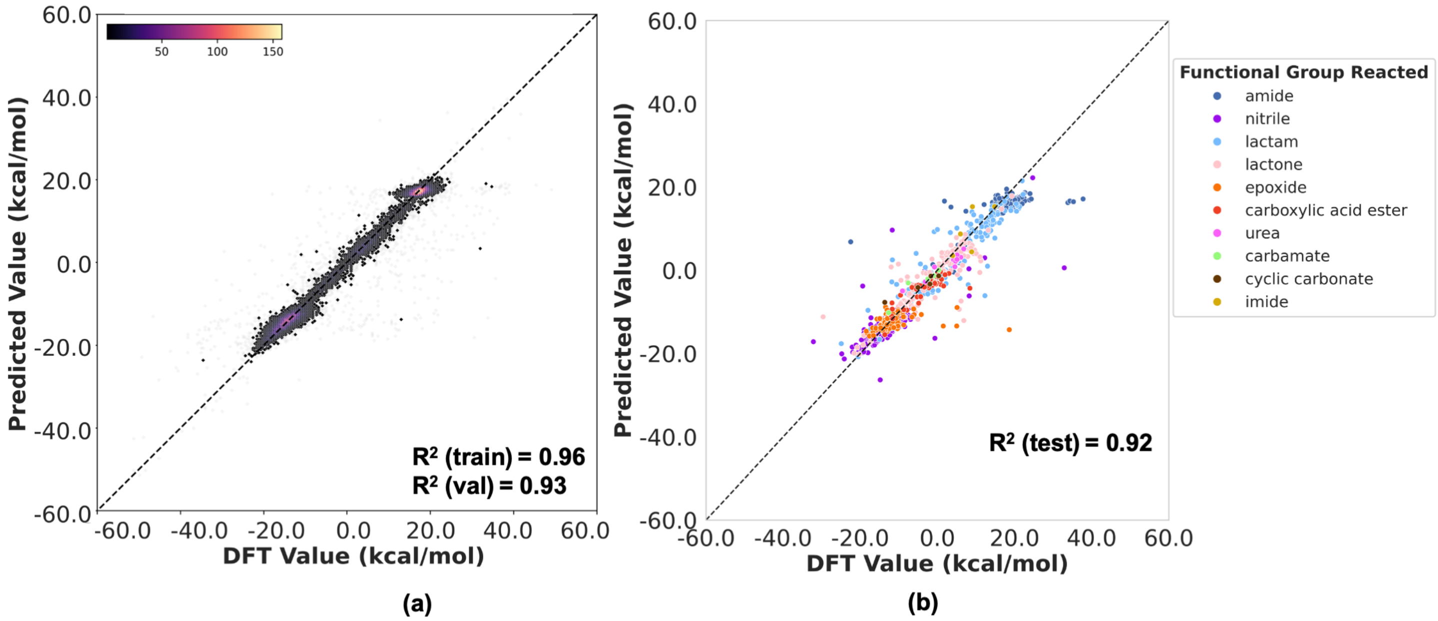

Figure S6: Performance of HEPOM on the QM9 only dataset. (a) ΔG_r predicted by HEPOM versus DFT reference labels for the train and test sets; (b) parity plot for the holdout test set segregated on the basis of hydrolyzed functional group.

Section S7: Examples of extreme outliers in the test set

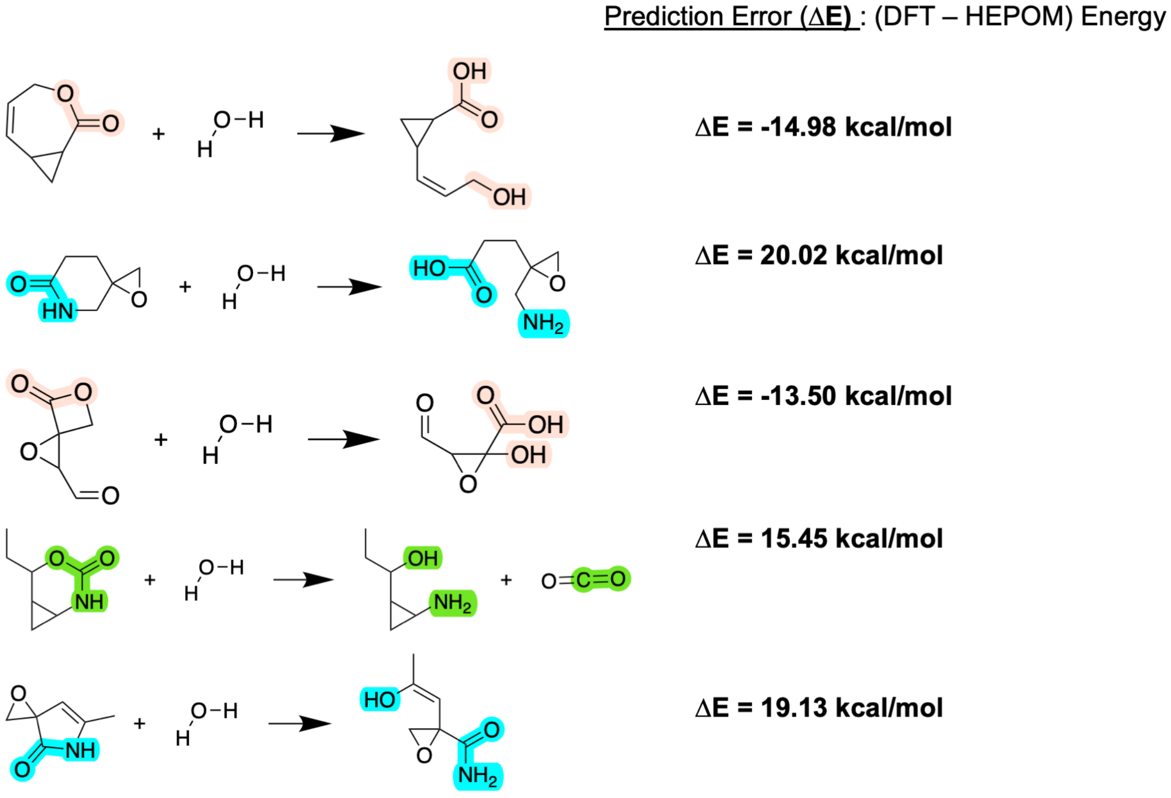

Figure S7: Outlier Reactions with high prediction errors.

Section S8: Evolution of the 2D feature embeddings with training epochs.

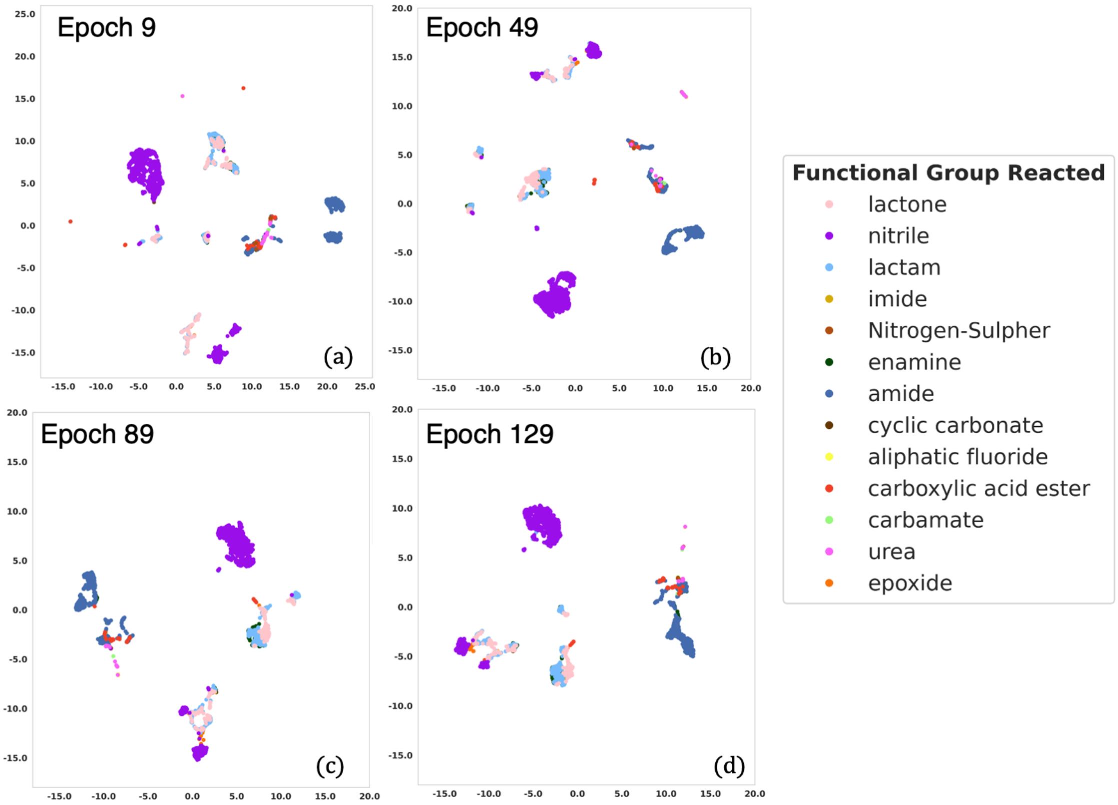

Figure S8: UMAP Embedding evolution during training of the high-dimensional feature vectors representing the hydrolysis reactions into a two-dimensional space

Section S9: Benchmark Comparison and Additional performance details for the QM9$^+$ and the QM9$^-$ datasets.

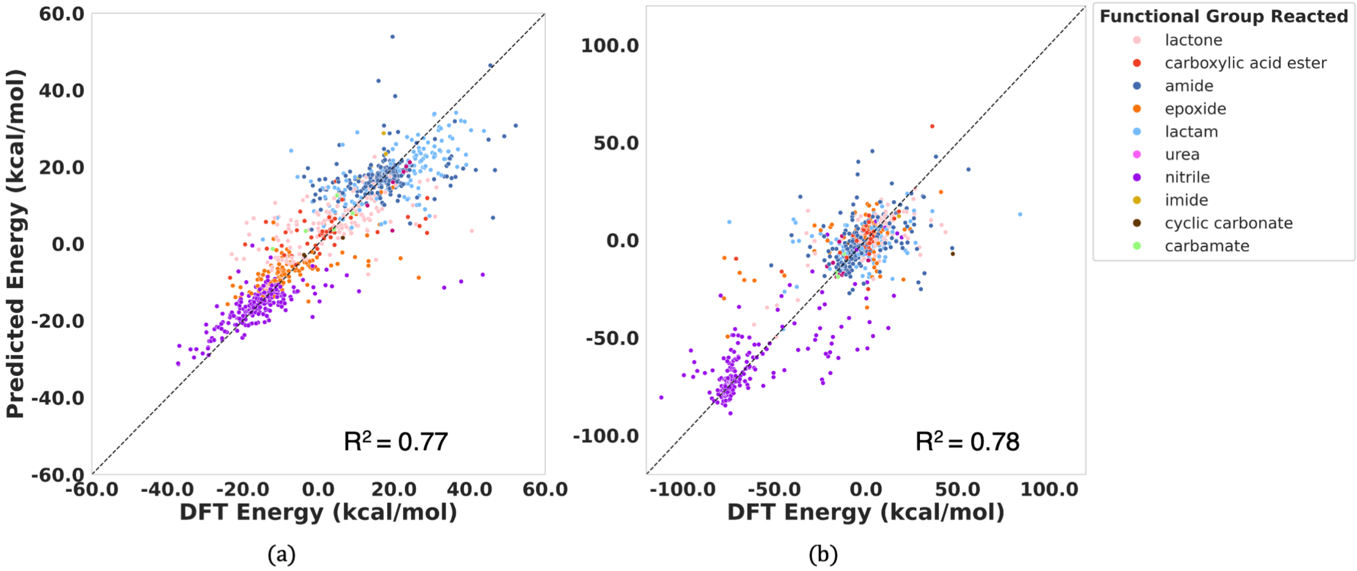

Figure S9(a): Parity plots for the performance of XGBoost+Morgan on the holdout test of the (a) QM9$^+$ dataset and (b) QM9$^-$ dataset.

Table S9(b): XGBoost MAE Statistics Based on Functional Group Hydrolyzed

**Functional Group**	`QM9`$^+$ **Test MAE (kcal/mol)**	`QM9`$^-$ **Test MAE (kcal/mol)**
Lactone	6.744	7.313
Nitrile	3.888	10.576
Lactam	7.197	9.663
Imide	7.407	5.124
Amide	5.301	7.554
Cyclic Carbonate	2.302	15.867
Carboxylic Acid Ester	6.871	6.834
Carbamate	5.304	2.751
Urea	4.556	7.786
Epoxide	4.541	9.273

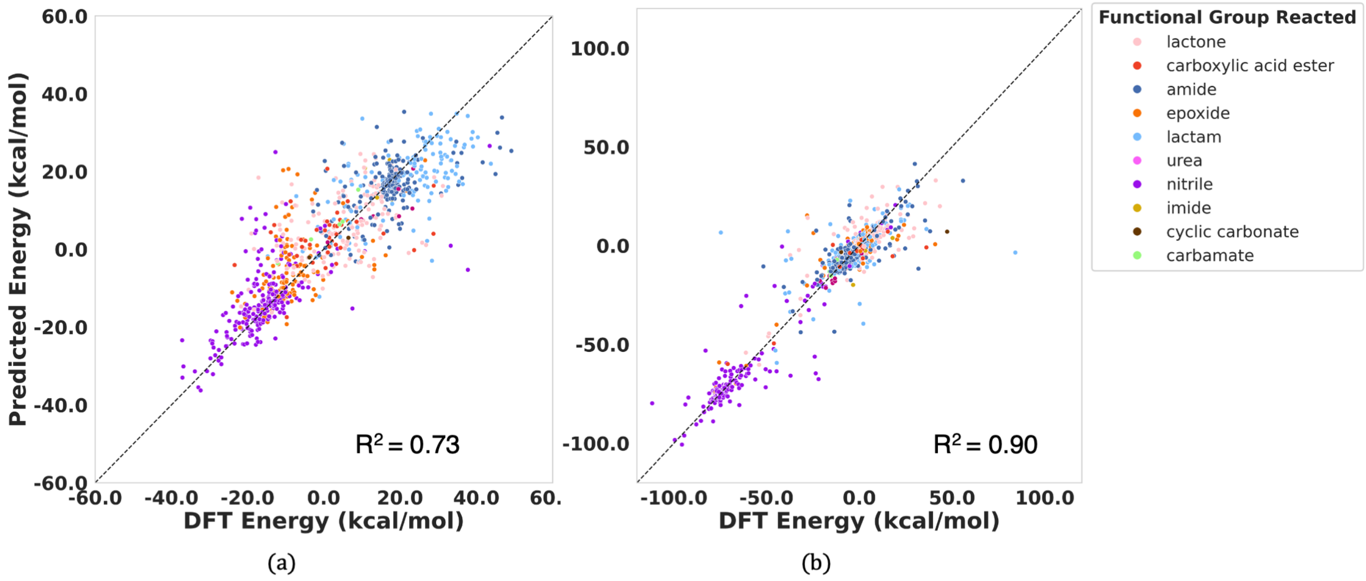

Figure S9(c): Parity plots for the performance of Chemprop on the holdout test of the (a) `QM9`$^+$ dataset and (b) `QM9`$^-$ dataset.

Table S9(d): Chemprop MAE Statistics Based on Functional Group Hydrolyzed

**Functional Group**	$\texttt{QM9}^{+}$ **Test MAE (kcal/mol)**	$\texttt{QM9}^{-}$ **Test MAE (kcal/mol)**
Lactone	7.873	5.974
Nitrile	5.283	4.890
Lactam	7.557	7.340
Imide	5.383	7.619
Amide	5.224	4.694
Cyclic Carbonate	1.851	16.926
Carboxylic Acid Ester	7.547	4.853
Carbamate	4.996	2.427
Urea	5.457	4.002
Epoxide	6.438	4.146

Table S9(e): HEPOM MAE Statistics Based on Functional Group Hydrolyzed

**Functional Group**	$\texttt{QM9}^{+}$ **Test MAE (kcal/mol)**	$\texttt{QM9}^{-}$ **Test MAE (kcal/mol)**
Lactone	4.706	5.596
Nitrile	4.079	10.393
Lactam	4.573	6.326
Imide	5.803	6.489
Amide	3.646	5.243
Cyclic Carbonate	2.277	16.756
Carboxylic Acid Ester	5.568	3.715
Carbamate	2.995	3.598
Urea	2.423	5.889
Epoxide	4.573	5.516

Section S10: Benchmark Comparison and Additional performance details for the combined datasets.

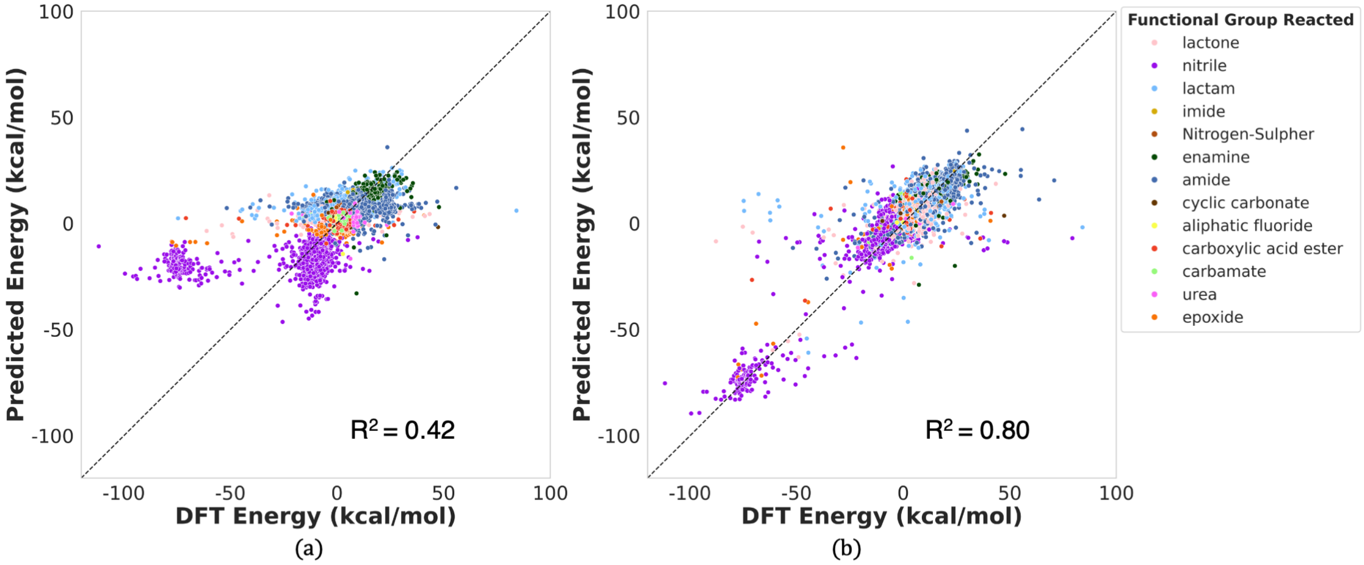

Figure S10: Parity plots on the combined holdout test for (a) XGBoost and (b) Chemprop.

Section S11: Model hyperparameters

Model	Global Pool	Loss Fxn	FC Hidden	Gated Hidden	Embedding Size	Dropout	Batch Norm
Protonated	Attention	MAE	[512, 256, 128]	[256, 256]	16	0.2	True
Neutral	Attention	MAE	[512, 256]	[256, 256]	12	0.2	True
Hydroxylated	Mean	MAE	[1024, 1024, 1024]	[256, 256]	16	0.2	True
QM9-Neutral	Mean	MAE	[512, 512, 512]	[256, 256]	16	0.2	True
Combined	Mean	MSE	[512, 256, 128]	[512, 512]	16	0.2	True

References

(1) Ong, S. P.; Richards, W. D.; Jain, A.; Hautier, G.; Kocher, M.; Cholia, S.; Gunter, D.; Chevrier, V. L.; Persson, K. A.; Ceder, G. Python Materials Genomics (pymatgen): A robust, open-source python library for materials analysis. *Computational Materials Science* **2013**, *68*, 314–319.
